# Significance of p85 expression as a prognostic factor for patients with breast cancer

**DOI:** 10.3892/ol.2014.2359

**Published:** 2014-07-18

**Authors:** WEIWEI ZHOU, GUANGYU AN, PING WEI, WENMING CHEN

**Affiliations:** 1 Department of Oncology, Beijing Chao-Yang Hospital, Capital Medical University, Beijing 100020, P.R. China; 2 Department of Pathology, Beijing Chao-Yang Hospital, Capital Medical University, Beijing 100020, P.R. China; 3 Department of Hematology, Beijing Chao-Yang Hospital, Capital Medical University, Beijing 100020, P.R. China

**Keywords:** breast cancer, p85, immunohistochemistry, prognosis

## Abstract

p85, the regulatory subunit of phosphatidylinositol 3-kinase (PI3K), functions in the pathogenesis and progression of human breast cancers. Previous studies have observed that p85 isoforms may correlate with cancer cell proliferation. In the present study, immunohistochemical staining of p85 was performed in 126 primary breast cancers. The association between the expression levels of p85 with clinicopathological variables, subtypes and prognosis was studied. The breast cancer specimens were divided into three subgroups according to the expression levels of p85 protein. High p85 protein expression was significantly correlated with tumor grade, vascular invasion and recurrence and/or metastasis (P<0.05). Increased p85 protein expression was associated with the human epidermal growth factor receptor 2-positive and triple-negative breast cancers (P=0.008). Patients with higher p85 protein expression levels showed shorter disease-free survival and overall survival times as compared with those with lower expression levels of p85 (P<0.001). Cox proportional-hazards analysis showed that p85 protein expression was not an independent prognostic factor. Further large-scale studies are required to evaluate the significance of p85 protein expression as a prognostic marker for breast cancer.

## Introduction

The phosphatidylinositol 3-kinase (PI3K) pathway functions in cell proliferation, migration and survival (1,2). Mutations of several components of the signaling pathway have been shown to lead to tumor progression in numerous cancer types, including glioblastoma (3), breast (4), ovarian (5), endometrial (6), lung (7) and thyroid (8). PI3Ks, major signaling hubs, are heterodimeric lipid kinases consisting of the p110 catalytic subunit and the p85 regulatory subunit, which is encoded by one of three gened; α, β and γ. p85 has two Src homology 2 (SH2) domains and an inter-SH2 domain that binds to the p110 catalytic subunit (1). The interaction between p85 and p110 has effects on the activity of p110, and results in alterations to downstream signaling.

Previous studies have reported the association between p85 isoforms and various cancers. Jaiswal *et al* (9) indicated that p85α mutants promote cell survival, Akt activation, anchorage-independent cell growth and oncogenesis. It was found that mutations in p85α abrogate its inhibitory effects on p110 from the stabilization activity, resulting in p110-dependent survival signaling. Sun *et al* (10) showed that expression of mutant p85 protein in chicken embryonic fibroblasts induced oncogenic transformation and increased proliferation. p85β expression has additionally been shown to be elevated in breast and colon carcinomas, and its increased levels correlate with PI3K pathway activation and tumor progression (11). p85α has been proposed to exert tumor suppressor properties based on observations in mice with a liver-specific deletion of the *Pik3r1* gene, which encodes p85 (12). It has also been demonstrated that inhibition of p85 activity by phosphopeptide 1257 (P-1257) delivery *in vivo* can significantly inhibit the proliferation of tumor cells (13). These studies may suggest that p85 is closely associated with tumor development, and may therefore be a potential target for therapeutic approaches. This previous research was preclinical and focused on cells or animals that did not show an association between p85 and cancer prognosis.

In the present study, p85 protein expression was analyzed by immunohistochemistry (IHC) in 126 primary breast tumors to elucidate the association between p85 expression and the prognosis of patients.

## Materials and methods

### Patients

One hundred and twenty six primary invasive breast carcinoma specimens were obtained from patients admitted between 2002 and 2005 to Beijing Chao-Yang Hospital, affiliated to the Capital Medical University of China (Beijing, China). The median age was 53 years (range, 27–84 years). A clinical history, treatment information and outcomes for each of the patients were obtained. Disease staging was performed according to the criteria of the American Joint Committee on Cancer (AJCC) TNM stage classification, seventh edition (2010) for breast cancer. Disease-free survival (DFS) was defined as the time from the date of diagnosis to the appearance of a regional recurrence or distant metastasis. Overall survival (OS) was defined as the duration from the date of diagnosis to the death of the patient due to breast cancer. The study was approved by the ethics committee of Beijing Chao-Yang Hospital, Capital Medical University (Beijing, China) and patients provided written informed consent.

### IHC and scoring

Immunohistochemical staining was performed by the immuno-bridge method in formalin-fixed paraffin tissue sections (4 μm). Sections were dewaxed in xylene and rehydrated through a graded alcohol series. Antigen retrieval was performed by placing the glass slides in EDTA (pH 9) at 98°C for 10 min under high pressure. The primary monoclonal rabbit antibody against human p85 protein (Zhongshan Golden Bridge Biotechnology Co., Ltd., Beijing, China) was incubated on the glass slides overnight at −4°C in a humidified chamber. The goat polyclonal polyperoxidase anti-mouse/rabbit immunoglobulin G (Zhongshan Golden Bridge Biotechnology Co., Ltd.) secondary antibody was then applied for 30 min at 37°C. Diaminobenzidine solution was used as a chromogen and the sections were counterstained with hematoxylin. Two pathologists independently assessed the staining results to determine the IHC score. p85 cytoplasmic staining was scored by multiplying the staining intensity score (0, 1, 2 and 3) by the percentage of stained cells (0–100%), to obtain the histochemical score (H-score; range, 0–300).

### Statistical analysis

Determination of the optimal p85 expression level cut-offs was performed using X-tile bioinformatics software (version 3.6.1, 2003–2005; Yale University, New Haven, CT, USA) (14). Statistical analysis was performed using SPSS 17.0 software (SPSS, Inc., Chicago, IL, USA). The association between p85 expression and the clinicopathological variables of the analyzed breast cancers was analyzed by the χ^2^ test. DFS and OS curves were calculated by the Kaplan-Meier method, and the log-rank test was used to evaluate the differences. A Cox proportional-hazards model was used to calculate the hazard ratio for each variable in the multivariate analysis. For all analyses, P<0.05 was considered to indicate a statistically significant difference.

## Results

### p85 protein expression

**Figure 1:**
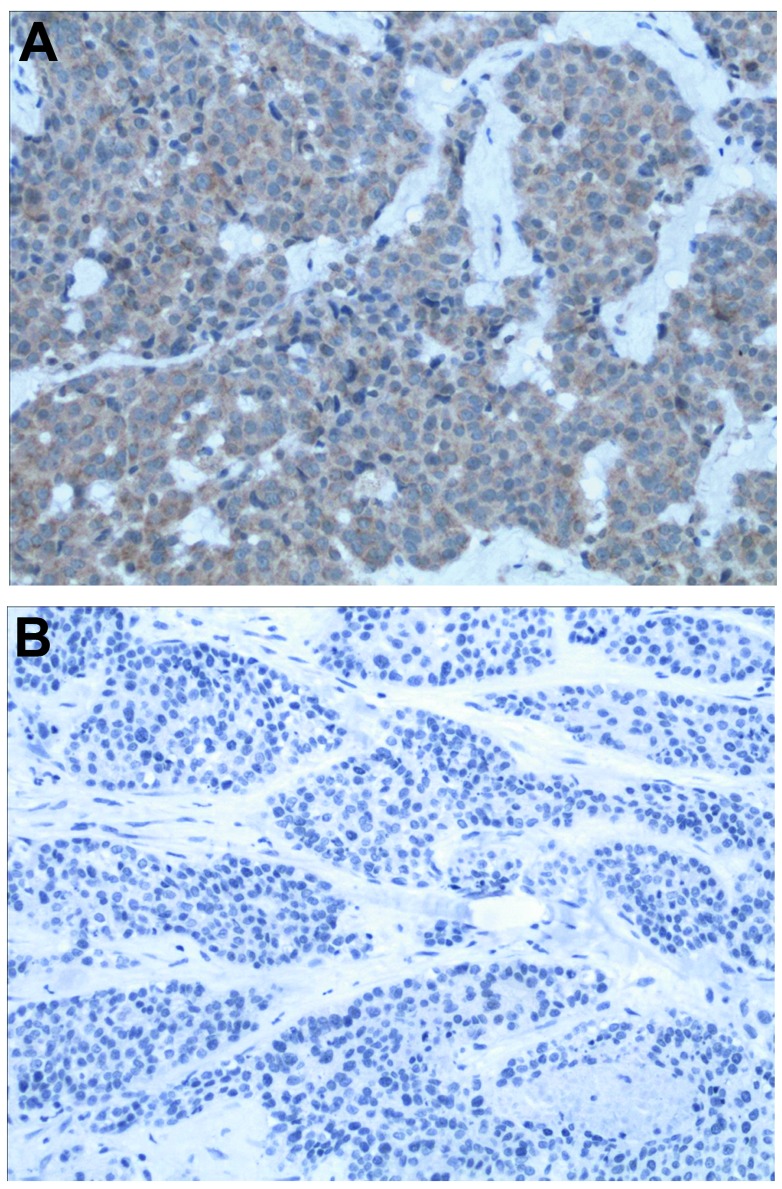
Immunohistochemical analysis of p85 protein expression in invasive breast cancer tissue. Diaminobenzidine solution was used as a chromogen and sections were counterstained with hematoxylin. (A) Positive and (B) negative p85 protein expression (magnification, ×200).

p85 protein expression in 126 breast cancer tissues was detected by IHC. The immunohistochemical staining showed that the expression was detectable in the cytoplasm of tumor cells (Fig. 1). Estrogen receptor (ER), progesterone receptor (PR) and human epidermal growth factor receptor 2 (HER2) status, as well as Ki-67 index, tumor size, tumor grade, lymph node status and vascular invasion status, were available from postoperative pathological reports.

The cut-off points were set using the X-tile bioinformatics software to divide the specimens into negative, moderately positive and strongly positive expression level subgroups. The optimal H-score cut-off points were 120 and 180, and the intervals of the three subgroups were 0–120, 121–180 and 181–300. The number of patients in each subgroup was 76 (60.3%), 28 (22.2%), and 22 (17.5%), respectively.

### Association between p85 protein expression levels and clinicopathological characteristics

The association between p85 protein expression levels and clinicopathological parameters is summarized in Table I. The expression levels of p85 protein were not correlated with patient age, menopausal status, clinical stage, tumor size, lymph node status or Ki-67 index. p85 protein expression levels were significantly higher in patients with a higher tumor grade, vascular invasion and recurrence and/or metastasis (P<0.05).

### Association between p85 protein expression levels and subtypes of breast cancer

The patients were classified into three subtypes according to the receptor status: ER- and/or PR-positive, HER2-positive and triple-negative (ER-, PR- and HER2-negative). The number of patients in each of these groups was 69 (54.8%), 25 (19.8%), and 32 (25.4%), respectively. As shown in Table II, p85 protein expression levels were significantly associated with breast cancer subtype (χ^2^=13.791; P=0.008). The proportions of moderately and strongly positive expression among the HER2-positive and triple-negative subtypes were higher as compared with the ER/PR-positive subtype.

### Association between p85 protein expression levels and survival

**Figure 2:**
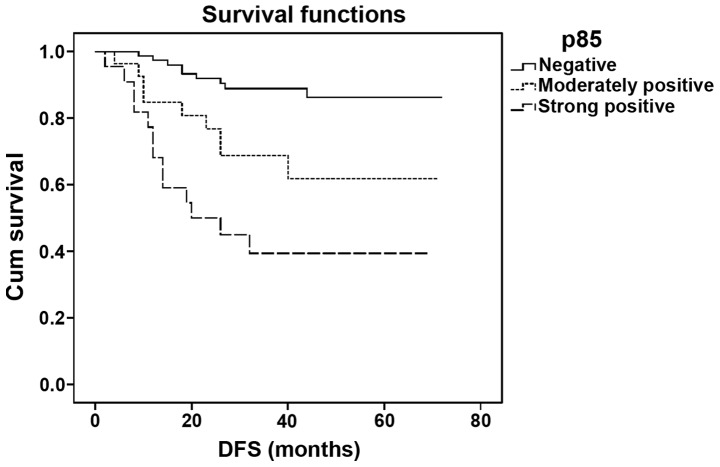
DFS time of patients according to the expression levels of p85 protein. DFS, disease-free survival; Cum survival, cumulative survival.

The median DFS time of the patients in this study was 34.5 months (range 2–72 months) and the median OS time was 40 months (range 5–72 months). Patients with higher p85 protein expression levels showed a shorter DFS as compared with those with lower expression levels (log-rank=28.078; P<0.001; Fig. 2). The DFS time of patients who were negative for p85 expression was significantly different from that of patients with moderately and strongly positive expression (P=0.006 and P<0.001, respectively). However, there was no significant difference between the groups with moderately and strongly positive expression (P=0.058).

**Figure 3:**
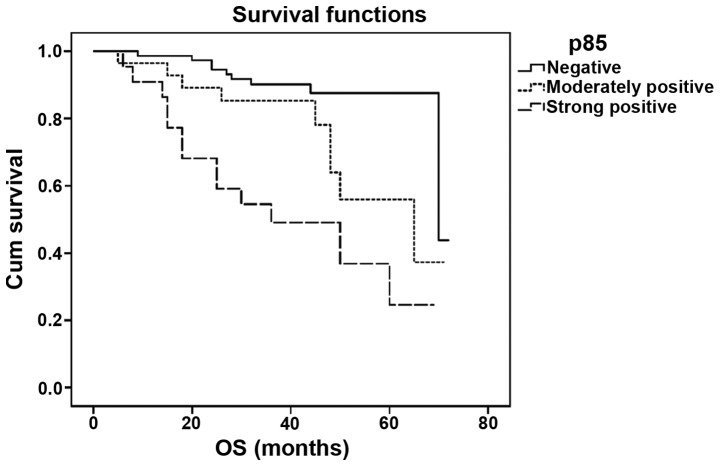
OS time of patients according to the expression levels of p85 protein. OS, overall survival; Cum survival, cumulative survival.

The OS time of patients with higher p85 protein expression levels was shorter than that of patients with lower levels (log-rank=26.043; P<0.001; Fig. 3), and the difference between each group was significant (P=0.023 for negative versus moderately positive; P<0.001 for negative versus strongly positive; and P=0.037 for moderately versus strongly positive). Cox proportional-hazards analysis, however, showed that p85 expression was not an independent prognostic factor in this model. The only variable correlated with survival was recurrence/metastasis (P<0.001).

## Discussion

Deregulation of the PI3K signaling pathway has been previously identified in breast cancer. Mutations to genes of the PI3K signaling pathway occur in >70% of breast cancers (15). The hyperactivation of the PI3K signaling pathway has been considered to promote resistance to current breast cancer therapies (15). A mutated form of the p85 regulatory subunit of PI3K has additionally been considered to be associated with hyperactivation of PI3K the pathway (10). In the present study, by using the X-tile bioinformatics software, p85 protein expression levels and the association with clinicopathological characteristics in breast carcinoma subtypes and the prognosis of patients, was investigated.

According to the H-scores of p85, patients in this study were divided into three subgroups: Negative, moderate, and strong positive expression level subgroups. The correlation between the PI3K p85 protein expression levels and the clinicopathological parameters were analyzed. The results indicated that the p85 expression levels were significantly higher in patients with a higher tumor grade, vascular invasion, and recurrence and/or metastasis. In a lung cancer study, the overexpression of p85 was demonstrated to correlate with the poor differentiation of primary lung cancer, and only weak or no expression was observed in the bronchial epithelial cells with phenotypic signs of metaplasia (17).

Breast cancer is a heterogeneous group of tumors and can be classified into subtypes according to ER, PR and HER2 status. Patients who are ER- and/or PR-positive are often considered to have a favorable prognosis, while patients with HER2-positive and triple-negative breast cancers (TNBCs) have a relatively poor outcome (18,19). In the present study, it was demonstrated that p85 expression levels were significantly associated with breast cancer subtype. Patients with the HER2-positive and TNBC subtypes of breast cancer displayed higher levels of expression of p85 than those with the ER/PR-positive subtype. The results suggested that the expression of p85 was different among the three subtypes of breast cancer.

Furthermore, it was demonstrated that patients with higher p85 protein expression levels had shorter DFS and OS times as compared with those with lower levels of p85 expression. This indicated that p85 may be a prognostic factor for patients with breast cancer. These findings were consistent with a previous observation in non-small cell lung cancer specimens, which suggested that high p85 expression was associated with poor survival (20). Patients with a strongly and moderately positive expression of p85 had a higher risk of mortality risk as compared with those with negative expression. However, there was no significant difference among the three groups.

In a previous study, the P-1257 inhibitor of p85 was administered to breast cancer cells *in vitro* and *in vivo,* and was found to possess strong potential to inhibit the PI3K pathway (13). This indicates that p85 may be a valid target for therapeutic intervention and can be utilized for the development of novel drugs.

In conclusion, p85 is a marker protein correlated with prognostic characteristics. p85 may serve as a predictive factor for patients with breast cancer, the inhibition of which may present as a useful therapeutic approach. However, further evaluation of the p85 inhibitor in breast cancer is warranted.

## Figures and Tables

**Table I:** p85 expression and clinicopathological characteristics.

	PI3K p85 expression, n (%)			
		
Clinicopathological parameter	Negative (n=76)	Moderately positive (n=28)	Strongly positive (n=22)	P-value^a^
Age, years				0.458^b^
<60	52 (60.5)	17 (19.8)	17 (19.8)	
≥60	24 (60.0)	11 (27.5)	5 (12.5)	
Menopausal status				0.730^b^
Premenopausal	15 (62.5)	4 (16.7)	5 (20.8)	
Postmenopausal	61 (59.8)	24 (23.5)	17 (16.7)	
Clinical stage				0.195^b^
I	25 (78.1)	4 (12.5)	3 (9.4)	
II	35 (56.5)	15 (24.2)	12 (19.4)	
III	16 (50.0)	9 (28.1)	7 (21.9)	
Tumor size, cm				0.334^b^
≤2	39 (67.2)	11 (19.0)	8 (13.8)	
>2	37 (54.4)	17 (25.0)	14 (20.6)	
Tumor grade^c^				0.004^b^
1	29 (82.9)	4 (11.4)	2 (5.7)	
2	35 (60.3)	12 (20.7)	11 (19.0)	
3	12 (36.4)	12 (36.4)	9 (27.3)	
Lymph node status				0.182^b^
Negative	41 (67.2)	13 (21.3)	7 (11.5)	
Positive	35 (53.8)	15 (23.1)	15 (23.1)	
Vascular invasion				<0.001^b^
No	69 (81.2)	14 (16.5)	2 (2.4)	
Yes	7 (17.1)	14 (34.1)	20 (48.8)	
Ki-67, n (%)				0.109^b^
<14	24 (70.6)	8 (23.5)	2 (5.9)	
≥14	52 (56.5)	20 (21.7)	20 (21.7)	
Recurrence/Metastasis				<0.001^b^
No	65 (75.6)	14 (16.3)	7 (8.2)	
Yes	11 (27.5)	14 (35.0)	15 (37.5)	

a P-values were derived from a comparison between negative, moderately positive and strongly positive subtypes;

b obtained using the χ^2^ test;

c according to the National Comprehensive Cancer Network cancer guidelines (16).

PI3K, phosphatidylinositol 3-kinase.

**Table II:** p85 expression and breast cancer subtypes.

	PI3K expression			
		
Parameter	Negtive, (n=76)	Moderately positive (n=28)	Strongly positive (n=22)	P-value^a^
ER/PR-positive, n (%)	50 (72.5)	13 (18.8)	6 (8.7)	0.008^b^
HER2-positive, n (%)	14 (56.0)	6 (24.0)	5 (20.0)	
Triple-negative, n (%)	12 (37.5)	9 (28.1)	11 (34.4)	

a P-values were derived from a comparison between negative, moderately positive and strongly positive subtypes;

b obtained using the χ^2^ test.

PI3K, phosphatidylinositol 3-kinase. ER, estrogen receptor; PR, progesterone receptor; HER2, human epidermal growth factor receptor 2.

## References

[1] Vivanco I, Sawyers CL (2002). The phosphatidylinositol 3-Kinase-AKT pathway in human cancer. Nat Rev Cancer.

[2] Bader AG, Kang S, Zhao L, Vogt PK (2005). Oncogenic PI3K deregulates transcriptionand translation. Nat Rev Cancer.

[3] Wang SI, Puc J, Li J (1997). Somatic mutations of PTEN in glioblastoma multiforme. Cancer Res.

[4] Sun M, Paciga JE, Feldman RI (2001). Phosphatidylinositol-3-OH-Kinase (PI3K)/AKT2, activated in breast cancer, regulates and is induced by estrogen receptor alpha (ERalpha) via interaction between ERalpha and PI3K. Cancer Res.

[5] Shayesteh L, Lu Y, Kuo WL (1999). PIK3CA is implicated as an oncogene in ovarian cancer. Nat Genet.

[6] Yokoyama Y, Wan X, Shinohara A (2000). Expression of PTEN and PTEN pseudogene in endometrial carcinoma. Int J Mol Med.

[7] Forgacs E, Biesterveld EJ, Sekido Y (1998). Mutation analysis of the PTEN/MMAC1 gene in lung cancer. Oncogene.

[8] Dahia PL, Marsh DJ, Zheng Z (1997). Somatic deletions and mutations in the Cowden disease gene, PTEN, in sporadic thyroid tumors. Cancer Res.

[9] Jaiswal BS, Janakiraman V, Kljavin NM (2009). Somatic mutations in p85alpha promote tumorigenesis through class IA PI3K activation. Cancer Cell.

[10] Sun M, Hillmann P, Hofmann BT, Hart JR, Vogt PK (2010). Cancer-derived mutations in the regulatory subunit p85alpha of phosphoinositide 3-kinase function through the catalytic subunit p110alpha. Proc Natl Acad Sci USA.

[11] Cortés I, Sánchez-Ruíz J, Zuluaga S (2012). p85β phosphoinositide 3-kinase subunit regulates tumor progression. Proc Natl Acad Sci USA.

[12] Taniguchi CM, Winnay J, Kondo T (2010). The phosphoinositide 3-kinase regulatory subunit p85alpha can exert tumor suppressor properties through negative regulation of growth factor signaling. Cancer Res.

[13] Folgiero V, Di Carlo SE, Bon G (2012). Inhibition of p85, the non-catalytic subunit of phosphatidylinositol 3-kinase, exerts potent antitumor activity in human breast cancer cells. Cell Death Dis.

[14] Camp RL, Dolled-Filhart M, Rimm DL (2004). X-tile: a new bio-informatics tool for biomarker assessment and outcome-based cut-point optimization. Clin Cancer Res.

[15] Miller TW, Rexer BN, Garrett JT, Arteaga CL (2011). Mutations in the phosphatidylinositol 3-kinase pathway: role in tumor progression and therapeutic implications in breast cancer. Breast Cancer Res.

[16] (2013). NCCN clinical practice guidelines in breast cancer.

[17] Lin X, Böhle AS, Dohrmann P (2001). Overexpression of phosphatidylinositol 3-kinase in human lung cancer. Langenbecks Arch Surg.

[18] Sorlie T, Tibshirani R, Parker J (2003). Repeated observation of breast tumor subtypes in independent gene expression data sets. Proc Natl Acad Sci USA.

[19] Foulkes WD, Smith IE, Reis-Filho JS (2010). Triple-negative breast cancer. N Engl J Med.

[20] Zito CR, Jilaveanu LB, Anagnostou V (2012). Multi-level targeting of the phosphatidylinositol-3-kinase pathway in non-small cell lung cancer cells. PLoS One.

